# Medical and Philosophical News

**Published:** 1784

**Authors:** 


### SECTION III.

### Medical and Philosophical News.

THE Academy of Sciences at Mantua have propoſed the following prize queſtion, viz. "In what conſiſts the poiſon of certain muſh-"rooms, and what are the ſureſt means of pre-"venting or remedying its effects?"—The prize

"is is to confift of two medals, each of the value of fifty florins. The differtations are to be fent to the Abbé Carli, fecretary of the Academy, before the firft of January 1785.

---

The following queftion is propofed by the Royal Academy of Sciences and Belles Lettres at Angers, for a prize of 300 livres, viz. "What "is the cheapeft method of bringing up found-"lings in the country, and what mode of edu-"cation is likely to render them moft ufeful to "the ftate?"—The differtations on this fubject muft be fent to M. de Narcé, fecretary to the Academy, before the firft of January 1785.

---

The Medical Society of Edinburgh have announced the following prize queftion, viz. "Quot fint aeris fpecies; quænam fingularum "natura, et in medicina vires?"—The premium is to be a gold medal of twenty guineas value. The differtations muft be written in Latin, and tranfmitted to the fecretaries of the Society before the firft of January 1785.

---

The

The Royal Academy of Surgery at Paris, at their public meeting on the 22d of April 1784, adjudged the premium for the queſtion announced laſt year (and which the reader will find in the 4th vol. of this Journal, p. 300) to M. Teſſier, ſtudent of ſurgery at Paris.—At the ſame meeting were read the eulogies of Meſſieurs de la Martiniere and Houſtet, two aſſociates lately deceaſed.—The ſecretary gave notice, that M. Vermont, a member of the Academy, and accoucheur to the queen of France, has founded an annual premium of 300 livres for the beſt paper on a midwifery ſubject that ſhall be preſented to the Academy in the courſe of the year.—The Academy have propoſed the following prize queſtion, viz. " In what caſes may probe ſciſ-

" ſars, which have been ſo much abuſed in com-

" mon practice, be employed with advantage;

" what are the reaſons which entitle them to a

" preference in particular caſes to other cutting

" inſtruments, and what are the different me-

" thods of uſing them?"—The premium is of 300 livres value, and the diſſertations muſt be ſent to M. Louis, ſecretary of the Academy, before Dec. 31, 1784.

---

Specimens of a new ſpecies of bark, the produce of the iſland of St. Lucia, and which promiſes to be a valuable acquiſition to the materia medica, have lately been ſent to the Royal Society. It is of the genus of *cinchona*, but of a different ſpecies from the *cinchona officinalis*. It exceeds the latter in bitterneſs and aſtringency, and is ſaid to have proved even more efficacious than the red bark, tho' given in much ſmaller doſes. In doſes, of more than twenty grains, it conſtantly excites vomiting.

---

An account of a very curious experiment to prove that water is a compoſition of dephlogiſticated and inflammable airs, has been lately communicated to the Royal Academy of Sciences at Paris by Meſſieurs Meunier and Lavoiſier. Theſe gentlemen recollecting the affinity which the calcinable metals are known to have with dephlogiſticated air, placed a tube of iron acroſs a furnace in ſuch a manner, that the tube might be made red hot. They then conveyed into the tube ſo heated the vapour of boiling water, and they found that its dephlogiſticated air was attracted by the iron, and that its inflammable air paſſed in a ſeparate and pure ſtate into a reſervoir prepared pared for its reception at the other extremity of the tube. The iron being by this procefs loaded with dephlogifticated air was found to be increafed both in bulk and in weight. The diameter of the tube was leffened, and it was obferved, that the iron was lefs attractable than before by the magnet. When the metal was in this manner faturated with dephlogifticated air, the decompofition of the water ceafed.—No fuch effects took place, when, inftead of a tube of calcinable metal, one of gold or filver was employed.—The inflammable air procured by this procefs was found to be ten or twelve times lighter than atmofpherical air.

---

Extract of a letter from Dr. Hunter of York, F. R. S. to Dr. Percival of Manchefter, F. R. S.

"On the 25th of October 1778, a fea-faring perfon, about forty years of age, was recommended as a patient to the Lunatic Afylum in York. During his abode in the hofpital he was never obferved to exprefs any defire for fuftenance, or to fhew any preference of it to his medicines. The firft fix weeks after his admiffion he was fed in the manner of an infant. A fervant undreffed him at night, and dreffed him in the morning; after which he was conducted to his feat in the common parlour, where he remained all day, with his body bent, and his eyes fixed on the ground. Every thing was indifferent to him, and he was regarded by all about him as an animal converted nearly into a vegetable. In this ftate of infenfibility he remained five years and fix months: but, on the 14th of May 1782, on his entrance into the parlour he faluted the convalefcents with the words *Good morrow to you all.* He then thanked the fervants of the houfe, in the moft affectionate manner, for their tendernefs to him, of which he had begun to be fenfible fome weeks before, but till then had not refolution to exprefs his gratitude. A few days after this unexpected recovery he was permitted to write a letter to his wife, in which he expreffed himfelf with becoming propriety. At this time he feemed to take peculiar pleafure in the enjoyment of the open air, and in his walks converfed with freedom and ferenity. On making enquiry concerning what he felt, during the fufpenfion of his intellectual and fenfitive powers, he replied, that his mind had been *totally loft*; but that about two months before fore his full reſtoration to himſelf he began to have thoughts and ſenſations, which, at firſt, ſerved only to excite in him fears and apprehenſions, eſpecially in the night-time. On the 28th of May 1782 he returned to his family; and has now the command of a ſhip employed in the Baltic trade." *See Moral and Literary Diſſertations lately publiſhed by Dr. Percival.*

---

Extract of a letter from Mr. George Bew, ſecretary to the Literary and Philoſophical Society at Mancheſter, to Dr. Simmons; dated Mancheſter, April 16, 1784.

" The laſt number of the London Medical Journal is now before me, with Mr. Henry's obſervations on freezing, and latent heat.—There is ſomething ſtill myſterious in the act of congelation.—During the winter I was amuſing myſelf with ſome experiments on that ſubject, and on vegetation. The cold was intenſe, and in a room, which was without fire, the thermometer ſunk to 28° of Fahrenheit. Some hyacinth bulbs, in ſmall glaſſes, which were adjacent to ſeveral thermometers, did not ſeem affected by the cold. The water continued unfrozen. I placed another glaſs with an equal quantity of water, and imimmerged in it a very well graduated thermometer of Dollond's. In ſome time the temperature was reduced to 26°; ſtill the water ſhewed no ſigns of congelation, even when agitated; but upon withdrawing the thermometer, and dropping a ſmall particle of ice into the water, it immediately froze, and became a ſolid maſs. On plunging the thermometer into the veſſel which contained the vegetating bulbs it roſe near two degrees. I muſt remark to you, that this happened when the temperature of the air was below the freezing point; for when it was above it, I did not diſtinguiſh much difference. Does not this prove, that living vegetables have a power of generating heat, in ſome degree, analogous to that of animals? Dr. Percival tells me, that ſome ſimilar experiments have been made by Mr. John Hunter, which were read at the Royal Society."

---

In the preſent ſtate of literary improvement, it is imagined, there are very few perſons of any deſcription, who have not frequent occaſion to lament the want of acceſs to a Library larger than their own, and furniſhed and regulated in a manner different from any public one which at preſent present exifts. Men of learning and fcience, whofe tafte or profeffion leads them to ftudy with peculiar care fome particular branch of knowledge, are often under the neceffity of confulting works fo coftly and expenfive, as to be far beyond the reach of moft private fortunes. Inftances of fuch works are numerous. Moft of the modern publications in Natural Hiftory, together with every later book of Plates, are examples of this kind; and as the value of fuch works depends upon the accuracy and beauty of the engraving, it is impoffible to fubftitute for them others which may have been executed with lefs care. The tranfactions of the different Learned Societies in Europe are of very great extent, and would form a library of themfelves; and yet there is no man of fcience who has not frequent occafion both to confult, and to ftudy them. If, in order to this, he fhould be under the neceffity of purchafing fuch works, he would not only fuffer the hardfhip of purchafing what is large and expenfive, but alfo what in a great meafure will be ufelefs to him, fince they comprehend the whole circle of fcience, and he is employed only in the cultivation of fome part of it.

With

With a view to remedy this inconvenience, a Society, entitled the LONDON LIBRARY SOCIETY, hath lately been inftituted. It is particularly intended, that this Library fhall contain thofe books of fcience, whether of our own or of other countries, which it is difficult for individuals to procure; and as its funds increafe, that it fhall contain every book of importance in fcience.

Any perfon may become a member of this Society by paying one guinea entrance and one guinea *per annum.*—The Library, which is fituated in Crane-court, Fleet-ftreet, is to be open on Mondays, Wednefdays, and Fridays, from Twelve to Three, and on Tuefdays and Thurfdays from Six to Nine; during which time, the members may confult any books, or fend for them to their own houfes.—A committee for regulating the affairs of the Society are to be elected annually on the laft Thurfday in June.—The committee appointed for the firft year, are the Reverend Dr. Kippis, the Rev. Dr. Rees, the Rev. Mr. Walker, Dr. W. Saunders, Dr. Crawford, Dr. Lifter, Benjamin Vaughan, Efq. and William Vaughan, Efq.

---

We feel much ſatisfaction in being able to inform the lovers of Natural Hiſtory in this country, that the Linnæan Library and Muſeum (conſiſting of the books, manuſcripts, and correſpondence; the *Herbarium*, inſects, ſhells, corals, and minerals of the celebrated Linnæus) are ſoon to be removed from Upſal to London. —This valuable collection having been offered for ſale ſince the death of the younger Linnæus, has been purchaſed by Mr. Smith of Norwich, now a ſtudent of phyſic at Edinburgh, for the ſum of nine hundred guineas.

